# Inguinal Lymph Node Metastasis as Sole Manifestation of Ovarian / Fallopian Tube Cancer: a Review of the Literature

**DOI:** 10.7150/jca.88863

**Published:** 2023-09-25

**Authors:** Sofoklis Stavros, Anastasios Potiris, Nikolaos Machairiotis, Alexandros Fotiou, Pavlos Zarogoulidis, Eirini Drakaki, Theodoros Karampitsakos, Christos Koratzanis, Spyridon Michalopoulos, Dionysios Vrachnis, Panagoula Oikonomou, Nikolaou Christina, Charalampos Charalampidis, Stamatios Petousis, Aris Ioannidis, Dimitris Matthaios, Ekaterini Domali, Peter Drakakis

**Affiliations:** 1 Third Department of Obstetrics and Gynecology, University General Hospital “ATTIKON”, Medical School of the National and Kapodistrian University of Athens, Athens, Greece.; 2 Pulmonary Department, General Clinic Euromedica, Thessaloniki, Greece.; 3 First Department of Obstetrics and Gynecology, Alexandra Hospital, Medical School of the National and Kapodistrian University of Athens, Athens, Greece.; 4 Department of Obstetrics and Gynecology, Medical School of Democritus University of Thrace, Alexandroupolis, Greece.; 5 Department of Clinical Therapeutics, National and Kapodistrian University of Athens, Medical School, Alexandra Hospital, Athens, Greece.; 6 Surgery Department, Democritus University of Thrace, Alexandroupolis, Greece.; 7 Pathology Department, University of Cyprus, Cyprus.; 8 2nd Department of Obstetrics and Gynaecology, Aristotle University of Thessaloniki, 541 24 Thessaloniki, Greece.; 9 Surgery Department, Genesis Private Clinic, Thessaloniki, Greece.; 10 Oncology Department, General Hospital of Rhodes, Greece.

**Keywords:** inguinal lymph node, ovarian cancer, fallopian tube cancer, distant metastasis

## Abstract

**Background:** Ovarian/fallopian tube cancer is the deadliest gynecological malignancy. Most cases are diagnosed at an advanced stage, typically after the cancer has spread to the peritoneal cavity, or via lymphatic drainage. The presence of distant lymph node metastasis in the inguinal region is a rare manifestation of lymphatic metastasis. Since the 2014 FIGO staging revision, ovarian cancer patients with inguinal metastasis are classified as stage IVB. However, the clinical significance of such an upstaging remains under investigation.

**Materials and Methods:** Both Scopus and PubMed / MEDLINE databases were utilized, by inputting the following combination of keywords: (Ovarian cancer OR Fallopian tube cancer) AND (Inguinal lymph node AND Metastasis) on June 31^st^, 2023. The time of publication and text availability were not considered when searching the databases and all relevant articles in English were initially accepted.

**Results:** Twelve patients from equal number of case reports were included in our review. Mean age of diagnosis was 56,5 years old, with 3 out of 12 women to be premenopausal at the time of diagnosis. Regarding the histologic type, 67% (8 out of 12) of the cases were serous adenocarcinoma and 4 patients (33%) were diagnosed with fallopian tube malignancy. All patients, except one, were treated with primary cytoreductive surgery. In all patients optimal cytoreductive surgery was achieved. All patients, except one, received adjuvant chemotherapy. Regarding the disease-free survival, mean DFS is calculated approximately at 2 years (23,1 months).

**Conclusion:** Inguinal lymph node metastases from ovarian / fallopian tube malignancy as initial site of metastasis is extremely rare. However, patients with inguinal masses should be investigated for ovarian / fallopian malignancy. Further investigation ought to be conducted to enlighten the pathway and the oncological significance of inguinal lymph node metastasis in ovarian cancer patients.

## Introduction

Epithelial ovarian/fallopian cancer remains a highly dangerous form of cancer in women, ranking third in incidence and second in mortality among all gynecological malignancies, according to data from GLOBOCAN 2020. In 2020, approximately 313,959 new cases of epithelial ovarian cancer were reported worldwide, leading to 207,252 deaths 1.

Sadly, the majority of cases are diagnosed at an advanced stage, typically after the cancer has spread to the peritoneal cavity, resulting in a 5-year survival rate of only 30.8%. For patients with advanced-stage disease, treatment options include upfront cytoreductive surgery combined with platinum-based adjuvant chemotherapy, or neoadjuvant chemotherapy with bevacizumab followed by interval debulking surgery. In all cases, the main treatment objective is complete cytoreduction, meaning the removal of all visible tumor tissue within the peritoneal cavity after surgery. This has been identified as the most critical prognostic factor in advanced-stage ovarian cancer 2.

The most prevalent symptoms of ovarian cancer often include abdominal pain, distension, early satiety, and vaginal bleeding, either individually or in combination. Additionally, the primary sign frequently observed during the initial medical examination is the presence of a pelvic mass. It has been reported that some patients with ovarian cancer may also present with distant metastatic deposits in the cervix, vagina, or vulva at the time of their first medical visit.

Since 2014, according to the revised FIGO (International Federation of Gynecology and Obstetrics) staging system, women who have inguinal lymph node metastasis, which was previously categorized as stage IIIC, are now reclassified to stage IVB. 3 This update reflects a change in the staging criteria and helps provide a more accurate and comprehensive classification of ovarian carcinoma, considering the presence of distant lymph node metastasis in the inguinal region. However, the occurrence of inguinal lymph node metastasis as the first and only manifestation of metastatic ovarian cancer is exceptionally rare in patients with early-stage ovarian carcinoma. The prognostic significance of this reclassification has not been fully investigated or studied yet. Several retrospective studies are hesitant regarding the oncological significance of inguinal lymph node metastases if optimal cytoreduction is achieved 4.

The objective of this study is to conduct a comprehensive review of the existing published literature concerning cases where inguinal lymph node metastases serve as the sole and initial manifestation of ovarian cancer. The aim is to assess and analyze the prognostic, clinical, and oncological importance of such metastases. By examining these cases and their outcomes, the study intends to shed light on the potential implications of inguinal lymph node involvement in ovarian cancer and its impact on patient prognosis and treatment strategies.

## Materials and methods

A systematic, electronic search was performed in Pubmed / MEDLINE and Scopus. The following keywords were used for the search: ((ovarian cancer) OR (fallopian tube cancer) AND ((inguinal lymph node) AND (metastasis))). Regardless the time of publication and text availability, all the articles with an English title and abstract were initially accepted. Through the initial research, 199 publications were retrieved. Published guidelines for Systematic Reviews and Metanalyses (PRISMA) were followed in the present systematic review. All kinds of manuscripts were included in the current review. Only patients with sole inguinal lymph node metastasis from ovarian/fallopian tube carcinoma and no other intraperitoneal/extraperitoneal metastasis were included in our study. Patients with no primary site detection of malignancy were excluded from our study.

This manuscript is a systematic review of the already published literature. Therefore, no institutional review board (IRB) approval was required.

From the initial 199 records identified, 41 duplicates were removed leading to 158 studies at initial screening. Title and abstract of all 158 publications were screened independently by two reviewers (A.P. and A.F.) and the full text of the eligible ones was screened subsequently. If there was a study selected only by one reviewer, the decision was taken by a third reviewer (N.M.).

**Figure 1:**
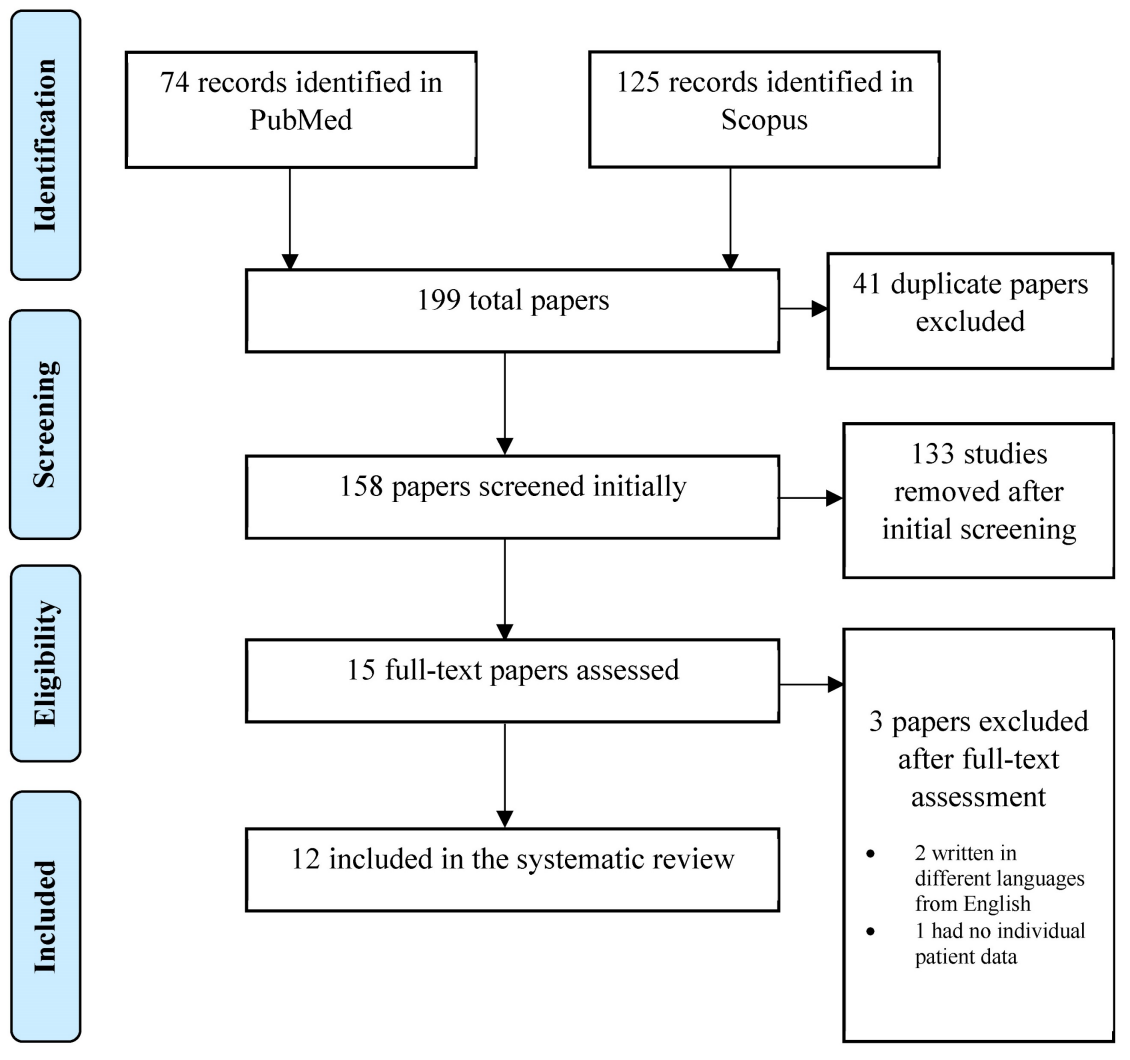
PRISMA Flow chart

After the initial screening, 133 studies were excluded because they did not conform to the inclusion criteria of the present review and 15 studies were selected for full-text assessment. When the full-text assessment was performed, two studies were not written in English language and one study lacked individual patient data regarding oncological management and disease-free survival (DFS). After a careful evaluation**,** twelve studies were selected for data extraction. The PRISMA flow diagram in **Figure 1** schematically presents the stages of article selection.

Data extraction was performed by a single reviewer (A.F.) and cross-checked by a second reviewer (A.P.). The data that was extracted included patients' characteristics such as age, tumor characteristics, oncological management (primary surgery/neoadjuvant chemotherapy), complications and oncological outcomes were retrieved from the included articles. Disease-free survival (DFS) was defined as the interval time patient is alive without any evidence of recurrence of the disease after the initial surgical treatment.

## Results

In total, 12 published articles and 12 patients were eligible and were included in our review 5-16. Two patients that meet the standards in order to be included in our study were excluded because of lack of details regarding patients' characteristics 17. **Table 1** demonstrates the included patients' characteristics.

Mean age of diagnosis was 56,5 years old, with 3 out of 12 women to be premenopausal at the time of diagnosis. Regarding the histologic type, 67% (8 out of 12) was serous adenocarcinoma. 4 patients (33%) were diagnosed with fallopian tube malignancy. In only 7 out of 12 patients, levels of CA-125 were documented. Interestingly in 25% of these patients, despite the FIGO IVB stage, levels of CA-125 were found to be normal (< 35 U/ml), while in only one patient levels of CA-125 were documented as more than 10-fold than normal levels. All patients, except one, were treated with primary cytoreductive surgery. In all patients optimal cytoreductive surgery was achieved. All patients, except one, received adjuvant chemotherapy. Regarding the disease-free survival, mean DFS is calculated approximately at 2 years (23,1 months).

## Discussion

Ovarian carcinoma comprises a collection of cancerous growths arising from various cell line origins and typically peaks in incidence around the age of 60. Although it ranks as the third most frequent gynecological malignancy, ovarian cancer is the deadliest due to its often advanced stage at the time of diagnosis. The late detection and unfavorable prognosis can be attributed to the absence of specific symptoms associated with ovarian cancer 2.

Ovarian carcinoma frequently exhibits metastasis through lymphatic dissemination, affecting approximately 14-70% of women diagnosed with the disease. The primary locations for lymphatic spread in ovarian cancer are the pelvic and paraaortic regions. The drainage route of ovarian cancer through the inguinal lymph nodes is relatively rare, and metastases to the groin area have been reported in up to 3% of women with this condition 18. The rarity of inguinal lymphadenopathy arising from ovarian cancer should lead the physician to seek for other more common causes at first. Inguinal lymph node enlargement can arise from various underlying pathologies. One common cause is localized infection, where bacteria or viruses infiltrate the surrounding area, prompting the lymph nodes to respond and become enlarged. Inguinal lymphadenopathy can also result from sexually transmitted infections, such as syphilis, gonorrhea, or herpes. Non-infectious etiologies include immune system disorders, like rheumatoid arthritis or lupus, which can trigger inflammation and lymph node enlargement. Additionally, certain cancers, particularly those affecting the genital, urinary, or lower gastrointestinal tracts, can lead to metastasis and subsequent inguinal lymph node involvement. Moreover, inguinal lymph nodes remain a common site of lymphatic spread of metastatic melanoma of the lower extremities. In some cases, the enlargement may be reactive due to the nearby site of the primary tumor, while in others, it might signify cancer spread. Given the diverse range of potential pathologies, a thorough medical evaluation is essential to accurately diagnose and address the underlying cause of inguinal lymph node enlargement.

Τhe revised FIGO (International Federation of Gynecology and Obstetrics) staging system in 2014 has made a significant change in the classification of inguinal lymph node metastasis in women. Previously categorized as stage IIIC, it is now reclassified as stage IVB under the updated system. Aim of this recategorization was a more accurate and comprehensive approach to staging ovarian cancer and a better insight into the disease's progression and treatment planning 3.

However, after this ovarian cancer FIGO staging system alterations several investigators and articles have demonstrated their concerns regarding the clinical and oncological impact. More specifically, based on a cohort of more than eleven thousand patients with stage III or IV ovarian cancer between 2004 and 2013 retrieved from the National cancer Institute's Surveillance Epidemiology and End Results (SEER) database, patients that were classified as ovarian cancer stage IVB based on their inguinal lymph node metastases had improved survival compared to other stage IV patients. Moreover, results of this analysis mentioned a comparable survival outcome among women with inguinal lymph node metastases and patients with positive pelvic and para-aortic lymph node metastases, concluding that based on these findings if patients are treated based on gynecological guidelines and if optimal cytoreductive surgery is achieved, reclassification of ovarian cancer patients with inguinal lymph node metastases into FIGO stage IVB, does not provide any clinical significance and should not be supported 4. Similar to these findings, other articles have questioned this reclassification. More specifically, Rosendahl et al and Paik et al in their retrospective studies did not achieve to detect any statistically significant difference in overall survival between patients classified as IVA or IVB 19, 20. A relatively newly published retrospective single institution cohort study by Chalif et al with 652 women with advanced ovarian cancer, including 18 patients with inguinal lymphadenopathy, confirms that inguinal lymph node metastasis did not affect clinical and survival outcome. Moreover, this retrospective study concluded that optimal cytoreduction in these patients with no gross residual disease, even if inguinal lymphadenectomy is needed, was associated with improved progression free survival 21.

Our review aimed to investigate the role of sole inguinal lymph node metastases from an ovarian/fallopian tube malignancy in patients without any other extraovarian/extrafallopian lesion. Finally, after meticulous study of the published literature only 14 cases with sole metastasis of ovarian/fallopian tube cancer in inguinal lymph nodes were identified. Moreover, only 12 of them were included in our review showing the rarity of this pathology. Only one patient was treated initially with neoadjuvant chemotherapy 12, while in all patients, that were treated with upfront cytoreductive surgery, optimal cytoreduction were achieved.

Interestingly, through the existing literature, there have been only three reported cases where contralateral inguinal lymph node metastasis was observed as the initial manifestation of an ovarian malignancy. The first case was documented by Shulman et al in 1953, nearly 50 years later in 2007 Ang et al reported the second case and in 2016 Haidopoulos et al reported the third case 5, 11, 14. Because these manifestations are extremely rare, the effects of this clinical presentation on the patients' prognosis and overall survival remain still unknown. None of these articles that mentioned contralateral lymph node metastasis managed to provide insight into the potential lymphatic pathway. These occurrences are exceedingly rare, underscoring the unusual and unique nature of such presentations in the context of ovarian cancer, needing further investigation and research about the way of spreading in the contralateral inguinal nodes. A possible lymphatic pathway that could lead to contralateral lymph node involvement could be through the contralateral round ligament of the uterus, a hypothesis that should be further investigated.

The mean disease-free survival for patients with inguinal lymph node metastases was found to be around 2 years. Interestingly, this survival period is slightly lower compared to the disease-free survival documented in the SEER (Surveillance, Epidemiology, and End Results) database for all ovarian cancer patients, regardless of their FIGO stage, which was reported as 2.54 years. This suggests that patients staged as IV due to inguinal metastases actually exhibit a superior or at least similar survival outcome compared to patients who are down staged to a lower FIGO stage. In other words, despite being categorized as stage IV due to inguinal metastases, these patients have a disease-free survival that is comparable or better than patients with lower-stage ovarian cancer. This finding emphasizes the importance of considering the specific characteristics and factors of individual cases when assessing the prognosis and survival outcomes for ovarian cancer patients with inguinal metastases 22.

## Conclusion

Ovarian cancer is the deadliest gynecological malignancy, often manifesting as an intraperitoneal disease. Nevertheless, there are rare instances where inguinal lymph node metastases can occur and serve as the initial symptom, even in the absence of metastasis in other locations. In cases where patients present with inguinal masses, it is important to include ovarian/fallopian tube malignancy in the list of potential diagnoses.

Early detection of these malignancies is of utmost importance for improving the patient's chances of survival. Therefore, all patients with inguinal masses should undergo a thorough gynecological examination to identify any underlying gynecological malignancy promptly. This approach ensures timely diagnosis and appropriate management, leading to better outcomes for the affected individuals.

Further clinical and experimental studies are necessary to investigate the pathway and the oncological significance of inguinal lymph node metastasis in ovarian cancer patients.

## Figures and Tables

**Table 1:** Patients' characteristics

Author (year)	Age	Histologic type	CA-125 (U/ml)	Way of treatment	Adjuvant therapy	DFS
Shulman et al (1953) 5	63	Cystadenocarcinoma	NR	Primary cytoreductive surgery	Radiotherapy	1 month
McGonigle et al (1991) 6	59	Endometrioid ovarian carcinoma	205	Primary cytoreductive surgery	Chemotherapy (6 cycles cisplatin and Cytoxan)	32 months
Cormio et al (1996) 7	67	Squamous cell fallopian tube carcinoma	NR	Primary cytoreductive surgery	Chemotherapy (cisplatin, doxorubicin, cyclophosphamide)	72 months
Scholz et al (1999) 8	43	Serous ovarian cystadenocarcinoma	NR	Primary cytoreductive surgery	Chemotherapy (paclitaxel, carboplatin)	3 months
Manci et al (2006) 9	58	Low grade serous adenocarcinoma	NR	Primary cytoreductive surgery	Chemotherapy (6 cycles carboplatin and paclitaxel)	24 months
Cho et al (2006) 10	72	Serous fallopian tube adenocarcinoma	51,7	Primary cytoreductive surgery	Adjuvant chemotherapy	5 months
Ang et al (2007) 11	59	Serous ovarian adenocarcinoma	215	Primary cytoreductive surgery	Adjuvant chemotherapy	12 months
Deka et al (2013) 12	35	Serous ovarian adenocarcinoma	412	Neoadjuvant chemotherapy and interval debulking surgery	Adjuvant chemotherapy	24 months
Yang et al (2014) 13	54	Low grade serous ovarian cystadenocarcinoma	< 35	Primary cytoreductive surgery	Adjuvant chemotherapy (6 cycles carboplatin and paclitaxel)	60 months
Haidopoulos et al (2016) 14	34	Endometrioid ovarian adenocarcinoma	NR	Primary cytoreductive surgery	Adjuvant chemotherapy	12 months
Nazari et al (2019) 16	57	High grade serous papillary fallopian tube carcinoma	< 35	Primary cytoreductive surgery	NR	NR
Maeda et al (2022) 15	77	High grade serous fallopian tube adenocarcinoma	36	Primary cytoreductive surgery	Adjuvant chemotherapy (6 cycles carboplatin and paclitaxel) and PARP inhibitor (niraparib)	9 months

NR: not referred

## References

[1] Sung H, Ferlay J, Siegel RL, Laversanne M, Soerjomataram I, Jemal A (2021). Global Cancer Statistics 2020: GLOBOCAN Estimates of Incidence and Mortality Worldwide for 36 Cancers in 185 Countries. CA Cancer J Clin.

[2] Colombo N, Sessa C, Bois AD, Ledermann J, McCluggage WG, McNeish I (2019). ESMO-ESGO consensus conference recommendations on ovarian cancer: pathology and molecular biology, early and advanced stages, borderline tumours and recurrent disease. Int J Gynecol Cancer.

[3] Mutch DG, Prat J (2014). 2014 FIGO staging for ovarian, fallopian tube and peritoneal cancer. Gynecol Oncol.

[4] Nasioudis D, Chapman-Davis E, Frey MK, Caputo TA, Witkin SS, Holcomb K (2017). Should epithelial ovarian carcinoma metastatic to the inguinal lymph nodes be assigned stage IVB?. Gynecol Oncol.

[5] Shulman A, Ratzan WJ, Izenberg D (1953). Primary ovarian cystadenocarcinoma: solitary metastasis to contralateral inguinal lymph node. Am J Obstet Gynecol.

[6] McGonigle KF, Dudzinski MR (1992). Endometrioid carcinoma of the ovary presenting with an enlarged inguinal lymph node without evidence of abdominal carcinomatosis. Gynecol Oncol.

[7] Cormio G, Gabriele A, Rota SM, Perego P, Cantu MG, Zanetta G (1996). Massive groin node metastasis as presenting sign of squamous cell carcinoma of the fallopian tube. Aust N Z J Obstet Gynaecol.

[8] Scholz HS, Lax S, Tamussino KF, Petru E (1999). Inguinal lymph node metastasis as the only manifestation of lymphatic spread in ovarian cancer: A case report. Gynecol Oncol.

[9] Manci N, Bellati F, Graziano M, Pernice M, Muzii L, Angioli R (2006). Ovarian cancer, diagnosed with PET, with bilateral inguinal lymphadenopathy as primary presenting sign. Gynecol Oncol.

[10] Cho J, Grumbine FC, Díaz-Montes TP (2006). Inguinal node metastasis as the initial presentation of primary fallopian tube cancer. Gynecol Oncol.

[11] Ang D, Ng KY, Tan HK, Chung AY, Yew BS, Lee VK (2007). Ovarian carcinoma presenting with isolated contralateral inguinal lymph node metastasis: a case report. Ann Acad Med Singap.

[12] Deka P, Shrivastava S, Barmon D, Kataki A, Sarma A (2013). Ovarian carcinoma in normal size ovaries with inguinal lymph node metastasis: a case report inhibitor-like mechanism. journal of Cancer Therapeutics and Research.

[13] Yang XJ, Zheng FY, Xu YS, Ou RY (2014). Ovarian cancer initially presenting with isolated ipsilateral superficial inguinal lymph node metastasis: a case study and review of the literature. J Ovarian Res.

[14] Haidopoulos D, Stavrou S, Domali E, Loutradis D, Rodolakis A, Drakakis P (2016). EP30.06: An unexpected and unusual finding: contralateral inguinal lymph node metastasis as the only manifestation of ovarian cancer. Ultrasound in obstetrics & gynecology: the official journal of the International Society of Ultrasound in Obstetrics and Gynecology.

[15] Maeda M, Hisa T, Matsuzaki S, Ohe S, Nagata S, Lee M (2022). Primary Fallopian Tube Carcinoma Presenting with a Massive Inguinal Tumor: A Case Report and Literature Review. Medicina (Kaunas).

[16] Nazari Z, Torabizadeh J, Geran T, Jafarpour H, Shamshirian A (2019). Primary Fallopian Tube Cancer: An Unusual Case of Inguinal Lymphadenopathy. Journal of Midwifery and Reproductive Health.

[17] Giri S, Shah SH, Batra K, Anu B, Jain V, Shukla H (2016). Presentation and Management of Inguinal Lymphadenopathy in Ovarian Cancer. Indian J Surg Oncol.

[18] Kleppe M, Kraima AC, Kruitwagen RF, Van Gorp T, Smit NN, van Munsteren JC (2015). Understanding Lymphatic Drainage Pathways of the Ovaries to Predict Sites for Sentinel Nodes in Ovarian Cancer. Int J Gynecol Cancer.

[19] Rosendahl M, Høgdall CK, Mosgaard BJ (2016). Restaging and Survival Analysis of 4036 Ovarian Cancer Patients According to the 2013 FIGO Classification for Ovarian, Fallopian Tube, and Primary Peritoneal Cancer. Int J Gynecol Cancer.

[20] Paik ES, Lee YY, Lee EJ, Choi CH, Kim TJ, Lee JW (2015). Survival analysis of revised 2013 FIGO staging classification of epithelial ovarian cancer and comparison with previous FIGO staging classification. Obstet Gynecol Sci.

[21] Chalif J, Yao M, Gruner M, Kuznicki M, Vargas R, Rose PG (2022). Incidence and prognostic significance of inguinal lymph node metastasis in women with newly diagnosed epithelial ovarian cancer. Gynecol Oncol.

[22] Kurta ML, Edwards RP, Moysich KB, McDonough K, Bertolet M, Weissfeld JL (2014). Prognosis and conditional disease-free survival among patients with ovarian cancer. J Clin Oncol.

